# The impact of craft creation practice on university students’ mental health: a moderated network analysis

**DOI:** 10.3389/fpubh.2025.1502506

**Published:** 2025-03-28

**Authors:** Xuejie Jiang, Xunzhou Lu, Meihe Pu, Xiaowen Li

**Affiliations:** ^1^Academy of Art and Design, Anhui University of Technology, Maanshan, China; ^2^College of Built Environment, University Technology MARA, Campus Puncak Alam, Puncak Alam, Malaysia; ^3^Handicraft College, Hubei Institute of Fine Arts, Wuhan, China; ^4^Sichuan Institute of Industrial Technology, Deyang, China

**Keywords:** mental health, dual-factor model, moderated network analysis, craft creation practice, university students

## Abstract

**Introduction:**

There has been relevant research on the effectiveness of craft creation practices (CCP) on the mental health of university students. However, little is known about the potential mechanisms affecting the network structure of mental health under the dual-factor model.

**Methods:**

This study employs moderated network analysis methods (MNM) to explore the reorganization of connections between various mental health indicators after craft creation practices. The study involved 353 university students from Anhui Province, who were randomly assigned to an intervention group (*n* = 185) or a control group (*n* = 229) to participate in an 8-week, 48-h craft creation practice.

**Results:**

The results showed changes in the network structure of different mental health variables after the intervention. Unlike the control group participants, social well-being was negatively correlated with depression and stress.

**Conclusion:**

This study supports the feasibility of using network approaches to analyze the connections between mental health indicators defined by a two-factor model and the contribution of craft creation practices to altering the complex relationship patterns between positive and negative psychological dimensions.

## 1 Introduction

Mental health issues among university students have become a focal point in the realm of global higher education. According to the study by Beiter et al., approximately 30% of college students reported experiencing moderate to severe depressive symptoms, and 25% of students exhibited significant anxiety symptoms (1). Additionally, loneliness is considered one of the important factors affecting the mental health of college students (2). Research indicates that adequate social support can effectively alleviate loneliness among college students, thereby reducing the occurrence of negative mental health issues such as depression and anxiety (3). On the other hand, loneliness has been found to be closely related to mental health status, with highly lonely students more prone to mental health problems (4). Valentina et al.’s study examines the combined influence of family and social environments on college students’ mental health. It suggests that adverse factors within the family environment, such as excessive parental control, family conflicts, or financial difficulties, may lead to increased emotional distress and psychological burden among college students. Furthermore, social pressures, particularly the COVID-19 pandemic in recent years, have not only heightened students’ uncertainty about the future but also contributed to social isolation, intensified academic pressure, and employment anxiety, thereby triggering psychological issues such as anxiety and depression (5, 6). Mental health not only affects students’ academic performance and social interaction abilities but also has profound implications for their career development and quality of life (7, 8). Therefore, maintaining and promoting the mental health of university students holds significant practical importance and social value (9, 10).

The mental health issues of university students are increasingly attracting extensive attention from society and academia. Numerous studies have explored methods to promote the mental health of university students (11). For example, psychoeducation and mental health courses are vital means to improve students’ mental health. These courses help prevent and alleviate mental health issues by disseminating mental health knowledge and enhancing students’ self-awareness and emotional regulation abilities (12, 13). University counseling centers provide individual counseling, group guidance, and crisis intervention services, aiding students in resolving psychological distress and enhancing their mental health levels (14, 15). Establishing social support systems is also crucial for improving the mental health of university students. By fostering good peer, teacher-student, and family relationships, students can receive emotional support and practical assistance, thereby strengthening their ability to cope with stress (2).

In recent years, craft creation practices (CCP) have gradually been recognized as an important non-pharmacological intervention to enhance university students’ mental health. Research shows that CCP, by providing opportunities for creative expression and emotional release, help improve students’ mental health (16, 17). Moreover, CCP help enhance social connections and support. Craft activities usually take place in group settings, providing a platform for communication and interaction, promoting peer support and a sense of belonging (4). These social supports are crucial for alleviating loneliness and improving mental health (3).

However, previous studies have three main shortcomings: First, most studies ignore positive mental health and only investigate negative mental health. Dual-Factor Model of Mental Health suggests that a pathological approach is insufficient to explain mental health; positive strengths and qualities are theoretically and practically significant in resisting mental illness (18, 19). The Dual-Factor Model of Mental Health, proposed by Keyes, posits that mental health encompasses not only the absence of psychological disorders but also includes individuals’ subjective well-being and the presence of positive psychological functioning (20). This model delineates mental health into two distinct yet interrelated dimensions: psychological disorders (negative factors) and mental well-being (positive factors). This dual perspective allows for a more comprehensive assessment of mental health, focusing not only on the presence or absence of psychological issues but also on overall life satisfaction and happiness.

There exists a close relationship between CCP and the Dual-Factor Model. Engaging in craft creation activities can promote mental health on multiple levels. Firstly, craft creation serves as a positive form of emotional expression, contributing to the enhancement of individuals’ subjective well-being and life satisfaction, thereby strengthening the positive dimension of mental health (21). Moreover, the focus and engagement involved in the craft creation process can effectively alleviate stress and anxiety, reducing the incidence of psychological disorders (22). Lastly, the sense of achievement and self-efficacy derived from completing creative works helps individuals establish a positive self-concept, further promoting mental health (23). Therefore, this study adopts the dual-factor model to construct mental health indicators.

Second, previous studies mostly use cross-sectional data and standard procedures (i.e., mean differences and effect sizes) (24–28). These methods cannot deeply understand the internal mechanisms by which CCP impact mental health. To address this, this study applies network methods (specifically, moderated network analysis methods, MNM) to compare group changes in mental health network structures between post-test and follow-up assessments. MNM, an emerging analytical method, shows great potential in mental health research. It combines network analysis and moderation effect analysis to explore complex interrelationships between variables and their changes under different moderating factors (29). This method can reveal not only the direct influencing factors of mental health but also identify potential moderating variables, providing a more detailed and dynamic understanding. Mental health is not a static state but a dynamic process. The MNM allows researchers to conduct longitudinal analyses, observing changes in mental health symptoms and moderating factors over time (30). This dynamic analysis can help identify mental health development trajectories and key turning points, providing data support for the design of long-term intervention strategies.

Thirdly, MNM demonstrates significant advantages over traditional statistical methods in psychological research, especially in exploring the impact of CCP on the mental health of university students. Traditional statistical methods such as regression analysis and analysis of variance typically assume that relationships between variables are linear and independent, which limits the understanding of complex interactions and multidimensional relationships. However, MNM can construct dynamic network structures among variables, allowing researchers to examine multiple variables and their interactions simultaneously, thereby providing a more comprehensive and detailed analysis (31). Furthermore, MNM exhibits greater flexibility and interpretative power when handling high-dimensional data and complex models. The mental health of university students involves multiple dimensions, such as emotional regulation, stress management, interpersonal relationships, and more. Traditional methods often require model simplification when dealing with numerous variables, potentially overlooking important interactions. In contrast, MNM can comprehensively analyze the relationships between variables without simplifying the model, offering a deeper understanding. Finally, methodological advancements in MNM, such as stability testing and model comparison, enhance the reliability and reproducibility of results, which is particularly important in mental health research (29). These methodological advantages ensure the robustness of research findings and provide a solid foundation for subsequent studies.

Although the relationship between craft creation and mental health has been studied to some extent, much of the existing research primarily focuses on general psychotherapeutic methods or traditional mental health interventions. There is a lack of exploration into the unique mechanisms through which specific activities, such as craft creation, influence the mental health of university student populations. This study seeks to propose a comprehensive mechanism model to provide a more nuanced analytical perspective on the extent to which CCP modify the dynamic network structure of university students’ mental health as defined by the Dual-Factor Model. The dual-factor model of mental health consists of positive dimensions (emotional well-being, social well-being, and psychological well-being) and negative dimensions (depression, anxiety, and stress). We hypothesize that post-intervention measurements will reveal a tighter network structure within the intervention group, with stronger positive correlations within each dimension. This means that within both the positive and negative dimensions, the intervention is expected to strengthen positive connections. For the positive dimension, this might indicate that elements such as emotional well-being, social well-being, and psychological well-being become more mutually reinforcing. For the negative dimension, although it traditionally involves negative indicators, robust connections may suggest that the intervention helps participants manage distress symptoms more effectively, preventing them from exacerbating each other. Additionally, we anticipate more negative correlations between the two dimensions, implying that an increase in positive variables will lead to a decrease in negative variables, reflecting an overall improvement in mental health. We will use three-wave data—pre-intervention, post-intervention, and follow-up (2 months after the intervention)—to test our research hypotheses.

The choice of a two-month follow-up period is supported by multiple scientific justifications. First, a two-month follow-up interval effectively assesses the long-term effects of the intervention measures. While the initial effects of mental health interventions may manifest in post-tests, determining whether these effects persist requires verification through an extended follow-up period (32). The two-month interval provides ample time to observe the stability of the intervention effects. Second, a two-month follow-up period reflects the participants’ sustained engagement with craft creation activities in their daily lives. Improvements in mental health depend not only on participation during the intervention but also on participants internalizing and applying the learned skills in their everyday routines after the intervention concludes. A two-month timeframe is sufficient to observe this internalization process and its lasting impact on mental health (33). Third, existing literature supports the use of 2 months as a common follow-up time point in mental health intervention studies. Research indicates that this timeframe avoids data fluctuations caused by intervals that are too short and prevents interference from external variables that may arise with longer durations, thereby ensuring the scientific validity and reliability of the follow-up results (34). These methodological advantages ensure the robustness of the research findings and provide a solid foundation for subsequent studies.

## 2 Methods

### 2.1 Participants and procedure

Participants were 353 university students from Anhui Province, China. Further demographic data are provided in Table 1. From March 2024 to May 2024, the intervention group underwent lacquer painting art practice for a total of 48 h, conducted twice a week, with each session lasting 3 h. The control group did not receive any activity guidance or intervention. Measurements were taken 1 week before the intervention (pre-test), 1 week after the intervention (post-test), and 2 months after the intervention (follow-up). We used G*Power 3.1 software to estimate the sample size for this study. Based on an alpha level of α = 0.05, an effect size (*Cohen’s d*) of 0.50, and a statistical power of 80%, a minimum of 338 participants was required. The sample size used in this study met the required criteria.

**Table 1:** Sample characteristics of variables.

Variables		Control (*n* = 194)		Intervention (*n* = 159)		*t*
		Mean/%	SD	Mean/%	SD	
Age		21.82	2.34	20.74	1.59	
Sex						
	Male	44.22		42.63		
	Female	55.78		57.37		
Smoking						
	Current smoker	18.14		17.19		
	Current no smoking	81.86		82.81		
Drinking						
	Current drinker	17.22		16.86		
	Current no drinking	82.78		83.14		
Physical exercise						
	Regular physical exercise	47.01		47.73		
	No physical exercise	52.99		52.26		
T1 Emotional well-being		4.66	1.08	4.79	0.83	0.99
T1 Social well-being		3.85	1.03	3.89	1.03	0.25
T1 Psychological well-being		4.51	0.87	4.69	0.71	1.60
T1 Depression		0.76	0.67	0.82	0.70	0.57
T1 Anxiety		0.85	0.66	0.76	0.65	−0.98
T1 Stress		1.12	0.62	1.08	0.66	−0.35
T2 Emotional well-being		4.70	1.05	4.75	0.80	0.25
T2 Social well-being		3.90	1.00	3.95	1.05	0.20
T2 Psychological well-being		4.55	0.85	4.60	0.75	0.35
T2 Depression		0.80	0.68	0.49	0.62	−4.19***
T2 Anxiety		0.80	0.61	0.75	0.65	−0.50
T2 Stress		1.10	0.60	1.05	0.68	−0.40
T3 Emotional well-being		4.78	0.85	4.65	1.07	−0.11
T3 Social well-being		3.88	1.05	3.90	1.00	0.10
T3 Psychological well-being		4.60	0.80	4.72	0.75	0.15
T3 Depression		0.78	0.66	0.80	0.70	−0.15
T3 Anxiety		0.82	0.64	0.77	0.68	−0.25
T3 Stress		1.05	0.65	1.07	0.70	0.10

*p < 0.05; **p < 0.01; ***p < 0.001.

In this study, participants were allocated using a random number generation method. Firstly, all subjects meeting the inclusion criteria were assigned through a computer-generated random number table prior to the commencement of the experiment. Subsequently, participants were sequentially allocated to the experimental and control groups based on the ascending order of these random numbers. To ensure group balance, we conducted necessary reviews of the randomization results to confirm that there were no significant differences between the experimental and control groups in terms of basic variables such as gender and age. Additionally, the randomization process was carried out under double-blind conditions, meaning that both the participants and the researchers were unaware of the allocation process and group assignments. This approach was implemented to minimize the potential for selection bias to the greatest extent possible.

During the intervention phase, to ensure the blinding of the intervention process, the intervention courses were taught by experienced lacquer art instructors following a standardized curriculum. The instructors were not informed of the experimental design details of the study and conducted the courses strictly according to the standard teaching plan while avoiding discussions on mental health-related topics to minimize extraneous influences beyond the intervention factors. Additionally, the research team did not directly participate in the organization or implementation of the intervention courses; instead, independent course administrators managed the sessions. This design reduced direct interaction between researchers and participants, thereby lowering the risk of inadvertently disclosing group assignment information.

During the follow-up phase, all mental health assessment questionnaires were administered by independent evaluators who were unaware of the group assignments. These evaluators assigned questionnaires based solely on participant identification numbers and did not engage in any discussions or interpretations related to the intervention content to minimize potential influences on the measurement results due to researcher-participant interaction. All assessments were conducted in the same laboratory environment, with testing procedures consistent with those used in the pre-test and post-test phases, ensuring control over environmental variables that could affect the outcomes.

### 2.2 Measures

CCP in this study is exemplified by a lacquer painting art course, an 8-week face-to-face teaching project designed to enhance students’ artistic literacy and personal development through lacquer art creation. The course content includes the development and stylistic evolution of lacquer painting art, the material properties of lacquer materials, types of lacquer painting techniques, and the creation of sample works. Lacquer painting is a highly practical course that involves multiple crafts and creative processes, including the following aspects: (1) Base Preparation: The preparation of a suitable base, which can be wood, cloth, paper, etc., is crucial as the choice and treatment of the base significantly impact the quality and effect of the lacquer painting; (2) Lacquer Preparation and Coating: Lacquer needs specific processing and preparation to meet different usage requirements. The painting process usually involves multiple layers, each requiring drying and hardening under specific environmental conditions; (3) Decorative Techniques: Various techniques such as carving, inlay, and painting are used for decoration in lacquer painting; (4) Surface Treatment: After completing the basic coating and decoration, sanding and polishing are necessary to achieve a smooth surface effect. The entire creation process requires a high degree of patience and focus (see Supplementary material for Detailed Intervention Protocols). This study selected lacquer painting as an intervention method based on its dual advantages in psychological mechanisms and practical feasibility. From a psychological perspective, lacquer painting can effectively induce a flow state, as its highly focused creative process, challenge-skill balance mechanism, and immediate feedback characteristics contribute to enhanced emotional regulation, increased self-efficacy, and improved sense of social belonging (35). In terms of practical feasibility, lacquer painting is easier to organize and standardize in teaching compared to other crafts such as ceramics or sculpture. It has a lower technical threshold, higher safety, and greater operability, making it more suitable for implementation among non-art students in higher education settings.

The positive dimensions of mental health were assessed using the Chinese version of the Mental Health Continuum-Short Form (MHC-SF) (36), which evaluates emotional, social, and psychological well-being over the past month. The MHC-SF includes 14 items rated on a 6-point Likert scale (1 = never, 6 = every day). Sample items representing each subscale are: “How often did you feel happy?” (emotional well-being); “How often did you feel that you had something important to contribute to society?” (social well-being); and “How often did you feel that most aspects of your personality were good?” (psychological well-being). The Cronbach’s α reliability estimates for the MHC-SF during the three measurement periods were: emotional well-being (0.87, 0.86, 0.88), social well-being (0.82, 0.81, 0.80), and psychological well-being (0.79, 0.83, 0.84).

The negative dimensions of mental health were assessed using the Chinese version of the Depression, Anxiety, and Stress Scales (DASS-21) (37), which evaluates symptoms of depression, anxiety, and stress over the past week. The DASS-21 includes 21 items rated on a 4-point Likert scale (0 = did not apply to me at all, 3 = applied to me very much or most of the time). Sample items representing each subscale are: “I could not seem to experience any positive feeling at all” (depression); “I was worried about situations in which I might panic and make a fool of myself” (anxiety); and “I found it hard to wind down” (stress). The Cronbach’s α reliability estimates for the DASS-21 during the three measurement periods were: depression (0.84, 0.87, 0.89), anxiety (0.83, 0.85, 0.85), and stress (0.86, 0.89, 0.85).

### 2.3 Statistical analyses

To estimate group differences using the MNM, we employed the “mgm” R package, version 1.2-9 (38). Network visualization was achieved through nodes (variables) and edges (connections), where the width of the edges represents the strength of the connections. In our study, edges represent the conditional dependence between two variables after controlling for all other variables in the network. We used Gaussian Graphical Models, which indicate the unique associations between two variables after conditioning on the rest of the network (29). For instance, a negative connection between emotional well-being and depression suggests that individuals with high emotional well-being scores tend to have low depression scores, and this relationship cannot be explained by other variables.

The MNM allows for the use of mixed-type variables (Mixed Graphical Models, MGM) to fit the network, where one variable serves as the moderating variable for pairwise interactions between two nodes. We fitted a moderated MGM for each time point (pre-intervention, post-intervention, and follow-up), including a grouping variable with two categories (moderating variable) and six continuous variables (mental health indicators). The grouping variable (i.e., control group or intervention group) was introduced as a categorical moderating variable to compare group differences by conditioning on the moderating variable. The “condition” function in the “mgm” R package was used to condition on the grouping variable. We specified the values of the moderating variable as control group (1) and intervention group (2) at the three time points. The “mgm” package implements regularization parameters in the ℓ1-regularized nodewise regression algorithm. We selected regularization parameters through cross-validation, using a hyperparameter γ = 0.25 and the AND rule to combine estimates from the nodewise regressions. Cross-validation is preferred in small sample models due to its higher sensitivity in revealing results, though it carries the risk of lower specificity; that is, it has a higher probability of identifying true edges in the network but also a greater chance of including false edges.

To test the stability of the estimated parameters in the MNM, we used the “resample” function in the “mgm” R package, which obtains empirical sampling distributions through non-parametric bootstrapping. In our study, we applied 1,000 bootstrap samples. The “plotRes” function returns plots of the bootstrap sampling distributions for each pairwise and three-way interaction. Small variances in the sampling distributions indicate a stable network, while non-zero values with 95% confidence intervals that do not include zero suggest the potential presence of moderating effects in the network model. Analyses were conducted in R version 3.2.1.

## 3 Results

### 3.1 Descriptive statistics

The study participants consisted of 414 university students from Anhui Province, China. They were randomly assigned to either the intervention group (*n* = 185) or the control group (*n* = 229). The overall mean age was 21.28 years (*SD* = 1.84). Further demographic data can be found in Table 1. Responses from the intervention participants were included in the analysis only if they attended at least two-thirds of the sessions and completed the final project creation. Therefore, from the initial sample, only 159 participants from the intervention group and 194 participants from the control group were retained for further statistical analysis. For the participants who attrited, we conducted an attrition analysis to compare the differences in characteristics between those who completed the study and those who did not. The analysis results indicated that there were no significant differences between the completion group and the non-completion group in terms of demographic characteristics and study variables. This suggests that sample attrition did not introduce bias in these key characteristics of the study population.

### 3.2 Comparative analyses

Independent samples *t*-tests were conducted to compare the intervention and control groups at baseline, post-test, and follow-up. Before the experimental intervention, there were no significant differences in mental health between the intervention group and the control group, indicating that the participant allocation between the groups was random and the intervention was feasible. After the intervention, the depression scores of the intervention group were significantly lower than those of the control group (*t* = −4.19, *p* < 0.001), suggesting that the intervention may have reduced the depression levels in the intervention group. However, at the follow-up time point (T3), there were no significant differences between the intervention and control groups.

### 3.3 Network analysis

**Figure 1:**
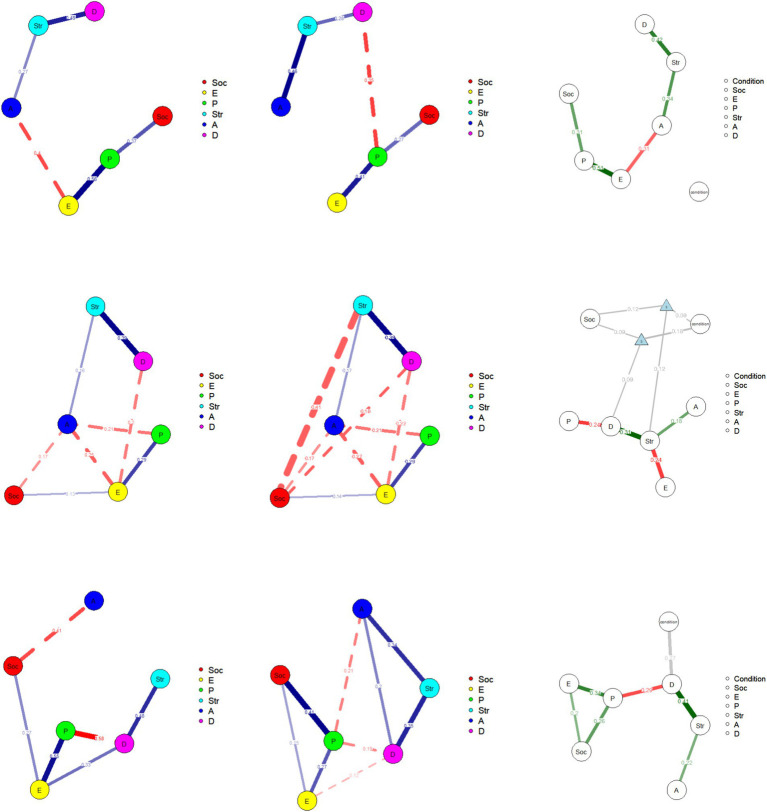
The results of the moderated network analysis. From top to bottom, the figures represent T1, T2, and T3. From left to right, they show the intervention group before the intervention, after the intervention, and at the follow-up. Soc, Social well-being; P, Psychological well-being; E, Emotional well-being; D, Depression; A, Anxiety; Str, Stress.

The visualizations of the MNM measured at each time point are shown in Figure 1. In the graphs, blue edges represent positive linear relationships, red edges represent negative linear relationships, and gray edges represent relationships involving the moderator. The moderation effects appeared only at Time 2. At each time point, the MNM’ visualizations are displayed in Figure 1. The moderation effects (three-way interactions) were observed only at Time 2, involving the variables of stress, depression, and social well-being (see Figure 1). Unlike the control group, in the intervention group, social well-being was negatively correlated with stress and depression. This suggests that the group condition moderated the impact of social well-being on stress and depression. Notably, the negative correlations between social well-being and both stress and depression disappeared over time.

### 3.4 Network robustness testing

**Figure 2:**
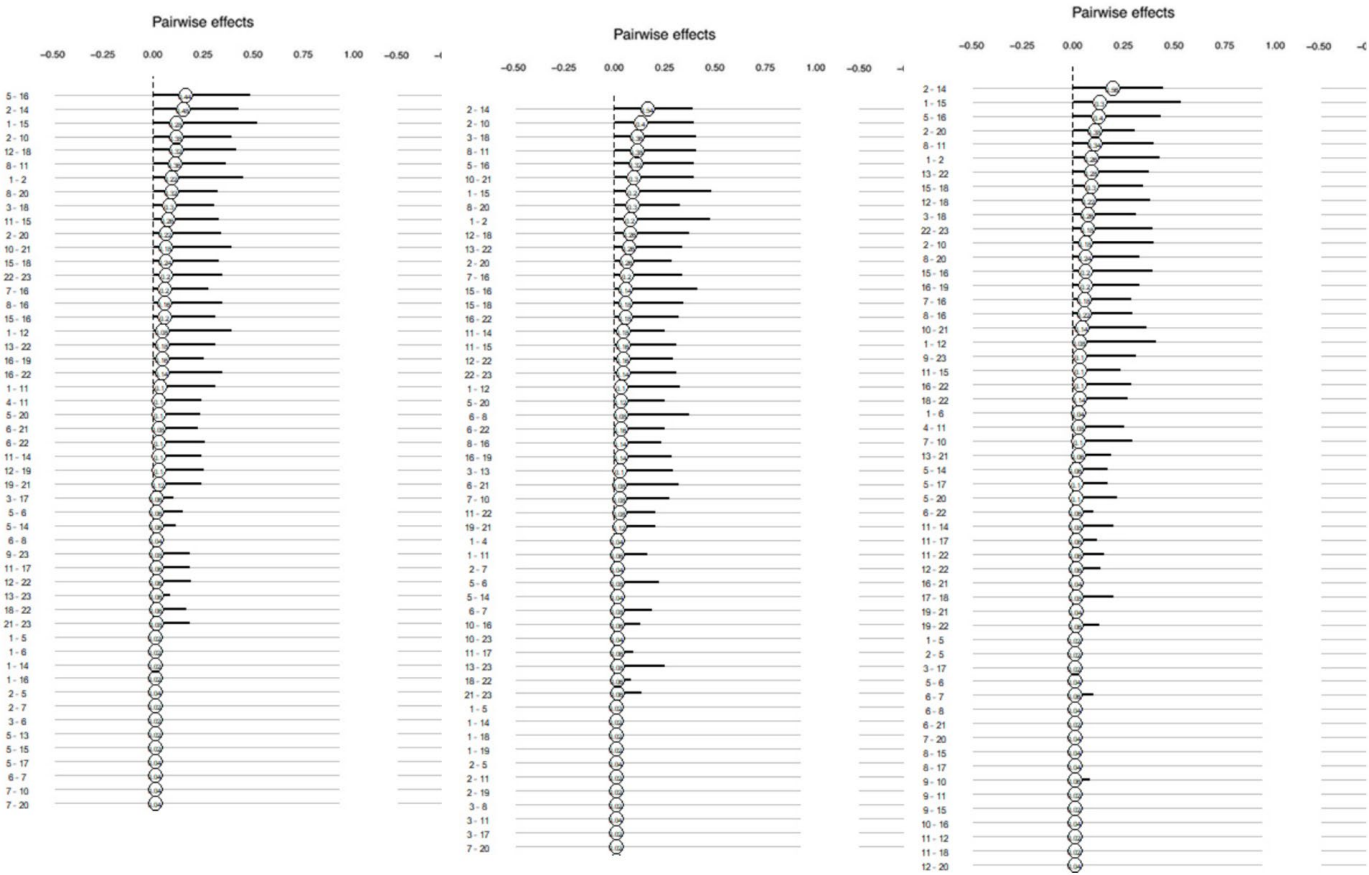
Bootstrapped sampling distributions at Time 1, 2, and 3 (in order).

Figure 2 shows the bootstrap sampling distribution of the MNM. The left panel displays pairwise effects, while the right panel shows moderation effects. The values represent the proportion of bootstrap samples where the given parameter estimates were non-zero, with lines indicating the 5 and 95% quantiles. The position of the values indicates the mean of the sampling distribution. Both pairwise effects and moderation effects were relatively stable over time, with the moderation effects being noticeably stronger at Time 2, as several values were significantly distant from zero. This indicates the robustness of the network model, particularly the moderation effects observed at Time 2.

## 4 Discussion

Traditionally, the dual-factor model conceptualizes mental health as a latent, unobservable variable characterized by high levels of well-being and low levels of depression. However, the estimated mental health network model in this study allows for a more detailed observation of the connections between these two dimensions. After the intervention, differences in the connections between these indicators were found between different groups.

First, the coefficients increased, indicating that the structure of the dimensions was more robust at Time 2 compared to Time 1. Second, compared to the control group, the intervention group exhibited a negative correlation between social well-being and both depression and stress. These findings highlight the role of social well-being, and the emergence of this indicator as central in the mental health network can be explained by flow theory.

Flow theory, proposed by Csikszentmihalyi, refers to a highly focused, immersed, and enjoyable psychological state experienced when individuals engage in an activity (39). The flow state is characterized by a high challenge-skill balance, where individuals feel a sense of efficacy, lose track of time, and experience enhanced pleasure. Flow not only aids in emotional regulation but also enhances social connections and well-being (40, 41).

Before the CCP, there was no significant correlation between social well-being and depression and stress among university students. This could be due to the lack of activities that effectively regulate emotions and enhance mental health. Under the high academic pressure and social expectations, university students are prone to anxiety and depression, with few effective coping mechanisms to manage these negative emotions (1). In this context, the role of social well-being was not evident. CCP significantly changed this situation by providing an activity that could induce a flow state. In the CCP, students needed to concentrate, using skills to meet challenges, all of which align with the characteristics of the flow state (21). Flow Theory posits that a flow state is a highly focused and immersive psychological state during activities, typically accompanied by a distorted sense of time and enhanced intrinsic rewards (21). Specifically, students enter a flow state in craft creation activities by setting clear goals, receiving immediate feedback, and finding a balance between challenges and skills. This deep concentration not only allows students to temporarily escape stressors from daily life but also promotes heightened focus and a sense of accomplishment. The flow state in craft practice promotes the generation and maintenance of positive emotions, helping individuals maintain psychological balance when facing academic and life pressures (42).

Flow experience plays a significant mediating role between CCP and mental health. Firstly, the flow state enhances self-efficacy, enabling students to face academic and life pressures with greater confidence and effectiveness, thereby reducing anxiety and depressive emotions (43). Secondly, flow experience fosters the generation of positive emotions and enhances students’ psychological resilience, allowing them to better cope with negative emotions (44). Furthermore, the deep concentration and sense of accomplishment experienced during flow states contribute to the enhancement of self-identity and social identity, further strengthening social well-being. Lastly, the craft creation activities in this study are conducted in a group environment, providing students with opportunities to interact with others and establish social support, thereby further enhancing their social well-being (45). Enhanced social support and a sense of belonging can effectively alleviate feelings of loneliness and social isolation, thereby reducing levels of depression and stress (46).

According to the therapeutic benefits of creative expression, CCP significantly enhance university students’ social well-being and mental health by promoting mindfulness and emotion regulation. Craft activities require individuals to fully concentrate on the current creative process, a state of high focus closely related to mindfulness (47). Mindfulness helps students reduce worries about the past or future, focusing on the “here and now,” thereby reducing anxiety and depressive emotions (48). Additionally, craft creation provides a safe outlet for emotion regulation, allowing students to process and release negative emotions through artistic expression (49). Research indicates that participating in craft activities can enhance emotion regulation abilities, reduce emotional fluctuations, and improve overall psychological resilience (50). In group craft activities, students not only gain personal accomplishment through creation but also build social support networks through interaction with others, further promoting social well-being. Therefore, CCP, through mechanisms of mindfulness and emotion regulation, not only improve individual mental health but also strengthen social connections and support systems, thereby comprehensively enhancing university students’ social well-being and mental health levels (51).

However, the moderating effect of CCP diminished over time, with both groups reporting the same mental health network structure in the follow-up assessment. At this point, the moderating effect of the group condition no longer existed, indicating that the longitudinal effects of CCP on the mental health network structure were weaker than the post-test effects.

Two month after the end of the CCP, the negative correlations between social well-being and both depression and stress disappeared, suggesting the temporality of the positive psychological effects brought by the CCP. To understand this phenomenon more deeply, several aspects can be considered: (1) Temporality of Flow Experience. The flow state is a highly focused and enjoyable experience, but its positive impact may be temporary. Studies have shown that the positive emotions and psychological effects brought by flow experiences gradually diminish after the activity ends (21). In this study, CCP provided college students with highly engaging artistic activities, allowing them to enter a flow state during the creative process, thereby enhancing social connectedness and mental well-being. During the intervention, participants experienced immediate satisfaction, a sense of belonging, and emotional release through their creations, leading to significant improvements in mental health. However, after the intervention ended, individuals returned to the pressures and challenges of daily life, and the positive effects of the flow experience gradually diminished. This explains why the negative correlation between social well-being and depression and stress disappeared 1 month after the craft practice. This finding also supports Csikszentmihalyi’s theory, which posits that a key characteristic of flow experiences is their dependence on specific environmental triggers. During the intervention phase, students received immediate feedback and interaction, which collectively facilitated the flow experience. However, after the intervention ended, students might have found it difficult to spontaneously maintain a similar environment in their daily lives, reducing the occurrence of flow experiences; (2) Reduction in Social Support. During the CCP, students establish good social relationships and support networks through joint participation in activities. However, this social support may not persist after the CCP ends. According to Bronfenbrenner’s ecological systems theory, individual development and mental health are influenced by multiple layers of environmental systems. Social support, as part of the microsystem, plays a crucial role in individual growth and mental health through sustained interaction and communication. Social support significantly impacts mental health, but it requires continuous interaction and communication to maintain its effectiveness (52, 53). After the CCP ends, the reduction in interaction and communication among students weakens the effectiveness of the social support network, leading to a diminished buffering effect of social well-being on depression and stress; (3) Fluctuations in Self-Efficacy. Self-efficacy refers to an individual’s belief in their ability to complete specific tasks. Through the creative process, CCP provides students with a sense of control and achievement, thereby enhancing their self-efficacy. During the intervention phase, students consistently received positive feedback, which strengthened their confidence in their abilities, subsequently increasing social well-being and reducing depression and stress. However, as the intervention ended and students returned to academic and social pressures in their daily lives, the absence of continuous positive feedback may lead to a decline in self-efficacy, weakening its protective effect on social well-being. Additionally, according to Vancouver et al.’s theory, self-efficacy generally requires ongoing challenges and successful experiences to be maintained (54). After returning to their regular academic routines, students may experience fluctuations in self-efficacy due to a lack of sustained motivation similar to CCP or the presence of new challenges and failures. This, in turn, may affect their social well-being. This also explains why the moderating effect of CCP was significant at Time 2 but weakened at Time 3.

To sustain the positive psychological effects of CCP and prevent its decline during the follow-up phase, a multi-level strategy can be adopted to consolidate intervention outcomes and promote long-term retention. Higher education institutions can integrate CCP into the curriculum by incorporating it as part of formal or elective courses, making it a stable component of students’ daily learning experiences. For example, CCP modules can be introduced in mental health education, art therapy, or creative thinking courses, combined with psychological theories such as mindfulness training and self-determination theory to help students derive emotional regulation and social interaction benefits during the creative process. With the increasing prevalence of online education, future research can also explore hybrid-mode CCP (incorporating both digital and offline components) to expand intervention reach and enhance its mental health benefits. Digital CCP can leverage online courses, VR/AR technology, and AI-driven personalized learning platforms to provide flexible remote creative experiences, allowing a broader range of learners to benefit. Meanwhile, hybrid-mode CCP can integrate online theoretical learning with offline practice, remote collaborative creation, and virtual-physical exhibitions, ensuring interactivity while improving accessibility.

This study has several limitations. First, although MNM provides an effective method for analyzing intervention data and exploring causal relationships between variables, we should remain cautious about the final causal interpretations. Despite the longitudinal design, establishing definitive causality remains challenging. Future research plans to use rigorous experimental designs to investigate causal relationships between variables. Secondly, this study involved 353 university students from a single province in China. The relatively small and geographically limited sample reduces the generalizability of the research findings. The applicability of the results to other cultural groups remains open to discussion. In this study, the collective creative process of CCP moderated the impact of social well-being on stress and depression. However, in Western cultures that emphasize individualism, mental health is more commonly understood as a state of self-actualization and emotional autonomy. Therefore, the psychological health effects of CCP may vary across different cultural contexts, necessitating further research to explore potential cross-cultural differences.

Third, this study focused only on individual-level psychological states without considering the group level. Given that mental health is influenced by complex social and environmental factors, future research could explore whether family members’ joint participation in CCP strengthens emotional bonds within the family, providing additional social support and thereby amplifying the intervention effects of CCP. Similarly, community-based CCP programs may enhance individuals’ sense of belonging and social cohesion, further promoting mental well-being. Future studies could draw on ecosystem theory (55) to examine the role of different levels of social interaction (e.g., family, community) in CCP interventions, providing a more comprehensive understanding of how social contexts influence the effectiveness of craft-based mental health interventions.

Fourth, according to Kozbelt et al., different types of craft practices may involve distinct psychological mechanisms. Craft activities can be broadly classified into two categories: one emphasizes individual focus and creativity, while the other relies more on group interaction and collaboration (56). In the former, individuals immerse themselves in the creative process and are more likely to experience a state of flow. In contrast, the latter may place greater emphasis on reducing loneliness and anxiety through the establishment of social support. Therefore, different types of crafts may influence mental health through different psychological mechanisms, and the findings of this study may not be applicable to all forms of craft practice. In future research, we will further compare the mental health effects of different types of craft activities.

Finally, in the current experimental design, the control group did not receive any specific activity guidance or intervention, which may result in changes in the mental health outcomes of the control group being influenced by other non-craft activities. Therefore, we plan to further optimize the design of the control group in future studies, such as by setting up a group of participants to engage in activities similar to those of the experimental group but not involving CCP. This approach will allow us to more accurately compare the effects of CCP, rather than changes merely due to participation in some activity.

## 5 Conclusion

This study confirmed the positive effects of CCP on mental health and, for the first time, employed MNA to thoroughly explore the impact of CCP on the network structure of the Dual-Factor Model of mental health, thereby unveiling the complex dynamic relationships among mental health indicators. Secondly, the research simultaneously examined both positive and negative psychological dimensions through the Dual-Factor Model and found that social well-being was negatively correlated with depression and stress in the intervention group, revealing the mechanism by which CCP alleviate negative emotions by enhancing social well-being. Additionally, the randomized controlled trial design and an appropriate sample size increased the reliability and empirical foundation of the results. Finally, this study found that social well-being became a central node in the mental health network after the intervention, supporting the crucial role of social connections in mental health interventions and suggesting that CCP may play a positive role in suicide risk prevention. The low cost, high accessibility, and creative expression characteristics of CCP highlight its significant potential for application in the field of mental health. Future research will further explore its role in suicide prevention, providing new perspectives and practical pathways for university mental health education, community psychological interventions, and public health policy.

## Data Availability

The raw data supporting the conclusions of this article will be made available by the authors, without undue reservation.
