# Supplementary material for: The impact of craft creation practice on university students’ mental health: a moderated network analysis
Source: Front Public Health. 2025 Mar 28;13:1502506. doi: 10.3389/fpubh.2025.1502506 (PMC11985433; doi:10.3389/fpubh.2025.1502506)
Supplement: Supplementary file 1 [file Supplementary_file_1.docx]

****Experimental Intervention Plan: A Case Study of Lacquer Art Practice Course****

***1.C****ourse Name***:** Lacquer Art

### *****2.Course Hours***:** 48 hours (8 weeks)

***3.****Assessment Methods and Grading Standards*****

The course assessment adopts a percentage-based system, combining formative assessments with a final evaluation. The course team establishes the assessment content and grading criteria.**Formative Assessment:** Based on the characteristics and training requirements of the "Lacquer Art Practice" course, a combination of attendance, class notes, regular assignments, and periodic tests will be used, accounting for 40% of the course assessment.**Final Assessment:** Students are required to submit a major course project as per the teaching requirements. The course team will collectively grade based on the grading standards, accounting for 60% of the course assessment.

***4.Textbooks and Main References***

**Textbook:** None

Reference Books:

(1)Explanation of Lacquer Decoration by Wang Shixiang, Life·Reading·Knowledge Sanlian Bookstore (July 2013)

(2) Red Lacquer Dreams: Chinese Ancient Lacquer Art by Qiu Cheng, China Bookstore (October 2012)

### *****5.Teaching Content*****

### This course is divided into the development and stylistic evolution of lacquer art; the material properties of lacquer; basic decoration techniques of the ten major lacquer crafts; tool making and material processing; types of lacquer decoration crafts and creative instance production, etc. Lacquer art is a highly practical course, combining art and craftsmanship. Through specific practical training, students master the basic craft techniques of lacquer, forming a method and language of artistic expression, ultimately achieving design thinking.

**Stage One: Fundamentals (Weeks 1-2)**

- Overview of Lacquer Art and Introduction to Tools and Materials
- Basic Techniques — Use of Lines and Colors
- Pattern Design and Composition Principles
- Creation of Basic Works and Feedback

**Stage Two: Technique Enhancement (Weeks 3-4)**

- Multi-layer Lacquer Techniques
- Creation of Special Effects (e.g., gold leaf, beads)
- Application of Mixed Media
- Mid-term Work Creation and Exhibition

**Stage Three: Creative Expression (Weeks 5-6)**

- Thematic Creation — Emotional Expression
- Free Creation and Personalized Expression
- Refinement of Works and Detail Polishing
- Group Work Creation and Collaboration

**Stage Four: Summary and Exhibition (Weeks 7-8)**

- Work Evaluation and Improvement
- Preparation for Final Exhibition
- Final Work Exhibition and Closing Ceremony

### *****6.Researchers*****

****Xunzhou Lu:**** Doctor of Arts, currently the head of the Lacquer Art Practice course. He possesses extensive experience in lacquer art creation and teaching, focusing on combining traditional lacquer techniques with modern artistic concepts. Dr. Lu has led and completed multiple national and provincial art projects and has exhibited personal and student works in domestic and international exhibitions, gaining wide recognition. In this study, he is responsible for the overall design and implementation, teaching basic and advanced lacquer techniques, guiding students in completing creative tasks, and providing professional art evaluations and feedback.

### ****Xuejie Jiang:**** Doctor of Art Psychology, with a profound research background in art psychology and rich experience in psychological counseling. Dr. Jiang has published numerous authoritative papers in the field of art and mental health, led multiple national research projects, and focuses on exploring the impact of art creation on mental health. In this study, she is responsible for experimental design and implementation, conducting mental health assessments and data analysis, and providing psychological support and intervention to students, ensuring the scientific and rigorous nature of the research methods.

***7.Applicable Majors:*** All students across the university

***8.Teaching Quality Monitoring:***

- **Teaching Plan Review:** Before the course begins, the teaching plan is reviewed by relevant internal departments to ensure compliance with teaching standards and experimental requirements.
- **Regular Inspections:** Monthly checks on course execution to ensure it follows the plan, with timely adjustments to teaching content and methods as needed.
- **Attendance Records:** Detailed records of each student's attendance to ensure high participation rates.
- **Assignment and Work Submission:** Students are required to submit assignments and works for each stage on time, with participation assessed based on completion.
- **Teaching Feedback:** After each stage, collect student feedback on course content, teaching methods, and instructor performance for analysis and improvement.
- **Achievements:** Organize student work exhibitions and exchange activities to evaluate the actual impact of art creation on students' mental health.

***9.Resources and Support:***

- **Materials and Equipment:** Provide high-quality lacquer art materials (such as lacquer, pigments, tools, etc.) and ample creative space.
- **Teaching Assistance:** Equip with multimedia devices for theoretical lectures and work presentations.
- **Expert Lectures:** Invite lacquer artists and psychology experts to conduct specialized lectures to broaden students' perspectives.
- **Field Trips:** Organize visits to lacquer art exhibitions or workshops to enhance practical experience.

***10.Expected Outcomes and Goals:***

- Students will complete at least two lacquer art pieces, demonstrating their mastery of techniques and creative expression abilities.
- Organize a work exhibition at the end of the course to promote communication and interaction among students.
- It is expected that through the course, students' stress levels will decrease, emotional stability will improve, and overall mental health will be enhanced.
- Quantify the specific impact of lacquer art practice on mental health through psychological assessment data.
